# Exploring mortality risk factors and specific causes of death within 30 days after hip fracture hospitalization

**DOI:** 10.1038/s41598-024-79297-z

**Published:** 2024-11-11

**Authors:** Cheng-Yi Wu, Ching-Fang Tsai, Yueh-Han Hsu, Hsin-Yi Yang

**Affiliations:** 1 Department of Orthopedics, Ditmanson Medical Foundation Chia-Yi Christian Hospital, Chiayi City, 600 Taiwan; 2 Osteoporosis Center, Ditmanson Medical Foundation Chia-Yi Christian Hospital, Chiayi City, 600 Taiwan; 3 Clinical Data Center, Ditmanson Medical Foundation Chia-Yi Christian Hospital, No. 539, Zhongxiao Rd., East District, Chiayi City, 600 Taiwan; 4 Department of Medical Research, China Medical University Hospital and China Medical University, Taichung, 404 Taiwan; 5 Division of Nephrology, Department of Internal Medicine, Ditmanson Medical Foundation Chia-Yi Christian Hospital, Chiayi City, 600 Taiwan; 6 Department of Nursing, Min-Hwei College of Health Care Management, Tainan, 736 Taiwan

**Keywords:** Hip fracture, Temporal trends, Mortality, Cause of death, Gender difference, Medical research, Risk factors

## Abstract

This study examined the sex-specific mortality risk and causes of death soon after hip fracture in Taiwan. The objective of this study was to evaluate the 30-day mortality risk and cause of death in patients with hip fracture and identify its associated risk factors. We conducted a retrospective cohort study using data from the National Health Insurance Research Database in Taiwan from 2000 to 2015. This study included patients aged 50 years or older who were admitted to a hospital with a hip fracture diagnosis for the first time. Of 285,891 patients hospitalized for their first hip fracture, 8,505 (2.98%) died within 30 days. The mortality risk of hip fracture declined from 3.76% in 2000 to 2.92% in 2015. Circulatory system diseases like ischemic heart disease and cerebrovascular disease were the most common cause of death and accounted for 22% of deaths within 30 days after fracture. Accidents and unintentional injuries caused 18% of deaths, followed by malignant neoplasms in 10% of deaths. Multivariate Cox regression identified older age, male sex, higher Charlson Comorbidity Index (CCI), suburban or rural residence, and lower income as risk factors for hip fracture mortality. The 30-day mortality risk of hip fractures has decreased but remains correlated with male sex, older age, and comorbidities. Circulatory diseases were the main cause of death. Early identification and treatment by experienced clinicians may optimize outcomes, minimize complications, and reduce mortality risk in the 30 days following hip fracture.

**Supplementary Information:**

The online version contains supplementary material available at 10.1038/s41598-024-79297-z.

## Introduction

Hip fracture is a major global public health concern, with an estimated 1.5 million annual cases worldwide^1^. This number is projected to increase to 2.6 million by 2025 and 4.5 million by 2050^2^. Hip fracture results in decreased functionality, a reduced quality of life, increased dependency, higher physical, mental, and economic burden, and elevated mortality risk. Despite advancements in surgical techniques and patient care, whether mortality rates are improving is unclear. Early mortality rates range from 5–10%^3^, whereas the mortality rate within one year after surgery can reach 8–36%^4–7^. Therefore, more research is urgently required to improve patient prognosis.

The causes of the high mortality rate after hip fracture are debated, with complications such as pulmonary embolism^8^, infection^9^, and heart failure^9,10^ potentially contributing. Factors related to falls and the risk of sustained osteoporotic fractures may also be involved^11^, along with low bone mineral density^12^. Additional analysis of mortality and its causes is crucial for identifying risk factors and predicting complications^13^.

Previous studies indicated cardiovascular disease and pneumonia as common causes of death after hip fracture^14–16^. However, the specific causes of death in the early postfracture period remain unclear. Given the projected increase in hip fractures, evaluating detailed information on specific causes of death within 30 days of fracture can help identify high-risk patients and enable personalized clinical care, optimize outcomes, and reduce mortality risk. This study aimed to analyze the 30-day mortality risk, causes of death and risk factors associated with death after hip fracture.

## Patients selection and methods

### Data source

 This study used data from the National Health Insurance Research Database (NHIRD) from 2000 to 2015. The NHIRD is the health insurance claims database for Taiwan’s National Health Insurance system. It encompasses demographic information, prescription drug usage, and disease records for more than 99% of the Taiwanese population. The National Death Registry contains data on primary and contributing causes of death and the date of death of all citizens. To protect privacy, all claim and registration files in the NHIRD are anonymized and given identification numbers. These anonymized data are available for academic purposes and provide valuable research opportunities. Our study identified diseases of interest using International Classification of Diseases, Ninth Revision, Clinical Modification (ICD-9-CM) codes. The NHIRD has been validated, with research articles based on it published in reputable scientific journals worldwide^17–20^.

### Ethics statement

The Institutional Review Board of the Ditmanson Medical Foundation Chia-Yi Christian Hospital, Taiwan (CYCH-IRB No: 2019063) approved this study, waiving the need for informed consent due to the anonymized nature of the data. All procedures adhered to pertinent guidelines and regulations, with full compliance ensured.

### Case definition

**Fig. 1:**
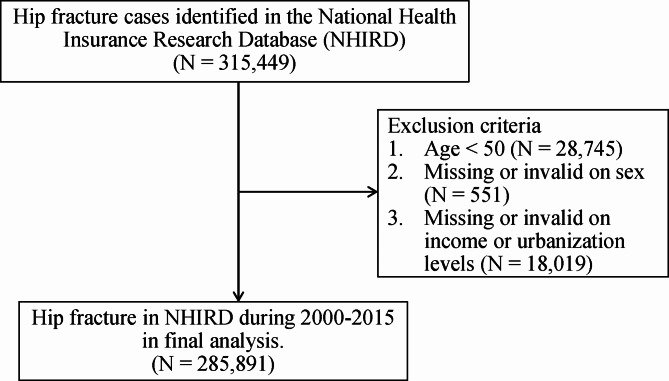
Flowchart showing patient selection in this study.

 We used the NHIRD to identify patients 50 years or older upon admission who had been diagnosed with their first hip fracture (ICD-9-CM: 820) between January 1, 2000, and December 31, 2015. Our analysis focused on inpatient cases and considered only the initial hospitalization records. Individuals with missing information on sex or age were excluded (Fig. 1).

### Mortality outcomes

We obtained information regarding the vital status, date of death, and cause of death of the study participants from the National Death Registry of Taiwan. We analyzed data on prespecified causes of death, including circulatory system diseases, malignant neoplasms, respiratory system diseases, infectious diseases, digestive system diseases, urogenital diseases, endocrine, nutritional, and metabolic diseases, accidents and unintentional injuries, and other causes. The other causes comprised deaths from specific categories, each accounting for < 3% of total deaths within a 30-day period. These included diseases related to the musculoskeletal system/connective tissue, the skin and subcutaneous tissue, the blood and blood-forming organs and certain disorders involving immune mechanisms, and the nervous system and sense organs; congenital malformations and chromosomal abnormalities; mental and behavioral disorders; intentional self-harm or suicide; and symptoms, signs, and ill-defined causes. The primary cause of death was determined by evaluating the ICD-9-CM codes, displayed in Supplementary Table S1. The data quality of the National Death Registry has undergone rigorous evaluation and is considered both valid and comprehensive, ensuring that the gathered information is reliable^21^.

### Covariates

The covariates included age, sex, the Charlson Comorbidity Index (CCI), urbanization, and income level. The CCI was calculated by assigning weights to the comorbidities^22^. The identification of comorbidities was based on the ICD-9-CM codes extracted from outpatient and inpatient reimbursement claims in the NHIRD. The comorbidity was defined based on diagnostic codes from at least two outpatient visits or one inpatient visit within 1 year prior to the hip fracture (defined as the index date). The CCI scores were categorized into four groups (scores of 0, 1, 2, and ≥ 3). Urbanization level was classified into three categories (urban, suburban, and rural), whereas economic status was divided into three groups based on the monthly insurable wage in New Taiwan dollars (NTD): <19,100 (low income level), between 19,100 and 41,999 (intermediate income level), and ≥ 42,000 (high income level)^23^.

### Statistical analysis

Continuous variables are reported as mean ± standard deviation and were compared using the t-test. Categorical variables are presented as percentages and were assessed using the Chi-square test for comparisons of categorical data. We examined the crude mortality rate per 100 people for all-cause mortality and the specified causes of death in the study population. Subgroup analyses were conducted based on sex, age, and year of hip fracture occurrence. To evaluate whether the 30-day mortality risk changed between 2000 and 2015, we conducted a joinpoint regression analysis using v.4.8.0.1 of the National Cancer Institute (US)^24^. This method identifies when changes in trends occur (inflection points). The dependent variable is a percentage, and the year of death is the independent variable. To test whether these inflection points were statistically significant and should be added to the model, four maximum inflection points and 4,499 Monte Carlo Permutation tests were conducted. The changes in the slope of these trends were calculated as the annual percentage change (APC), with a 95% confidence interval for each segment. This analysis identified periods with statistically different log-linear trends in 30-day mortality. In addition, we calculated the APC per period and the average APC over the entire study period. We also calculated standardized mortality ratios (SMRs) within 30 days. First, we collected the observed number of deaths within 30 days for our study population from Taiwanese mortality statistics. Then, we calculated the expected number of deaths by applying age-specific mortality rates from a reference population to the age distribution of our study population. The SMR was calculated as the ratio of observed to expect deaths. An SMR > 1 indicates excess mortality compared with the reference population, whereas an SMR < 1 suggests lower mortality. We conducted additional data analyses, including calculations of confidence intervals and subgroup analyses, to explore the SMR results further. A Cox proportional hazard model was also used to identify factors associated with 30-day mortality risk from hip fracture. All statistical analyses were conducted using SAS v.9.4 software (SAS Institute, Cary, NC, USA), and a significance threshold of 0.05 was set for two-tailed p-values.

## Results

### Characteristics of the study population

From January 1, 2000, to December 31, 2015, 285,891 patients experienced hip fractures. Among the patients, 8,505 (2.98%) died within 30 days of sustaining a hip fracture. Of these, 4,416 were men (51.92%) and 4,089 were women (48.08%). The patients who died within 30 days were older (79.51 ± 7.25 years) compared to the survivors (75.87 ± 8.99 years) (*p* < 0.001). They also had a higher CCI than survivors. Furthermore, significant differences in residence and income level were observed between the 30-day survivors and nonsurvivors (Table 1).

**Table 1:** Demographic characteristics among patients from 2000 to 2015.

	Alive after 30 days	Death within 30 days	*p*-value
Number	277,386	8,505	
Age, years	75.87 ± 8.99	79.51 ± 7.25	< 0.001
Sex			
Male	109,621 (39.52)	4,416 (51.92)	< 0.001
CCI			
0	48,712 (17.56)	952 (11.19)	< 0.001
1	132,248 (47.68)	3480 (40.92)	
2	52,476 (18.92)	1854 (21.80)	
≧3	43,950 (15.84)	2219 (26.09)	
Residence			
Urban	125,653 (45.30)	3490 (41.03)	< 0.001
Suburban	101,876 (36.73)	3246 (38.17)	
Rural or remote area	49,857 (17.97)	1769 (20.80)	
Income level			
Low	87,009 (31.37)	2738 (32.19)	< 0.001
Intermediate	170,190 (61.35)	5268 (61.94)	
High	20,187 (7.28)	499 (5.87)	

Data are presented as mean ± SD or number (percentage, %)

*CCI*  Charlson comorbidity index score.

### Trends in hip fracture mortality

**Fig. 2:**
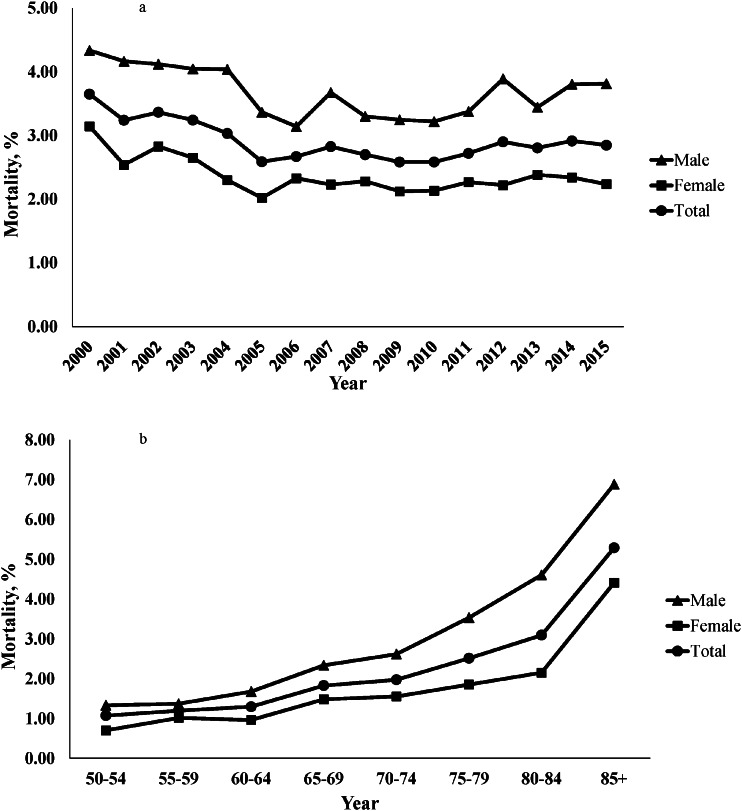
**a** Mortality rates of hip fracture for Taiwanese adults aged older than 50, stratified by sex and year (2000–2015); **b** age-related mortality rates of hip fracture in the overall population, females, and males.

The mortality risk within 30 days of hip fracture decreased from 3.76% in 2000 to 2.92% in 2015. The 30-day mortality risk initially trended downward from 2000 to 2006 (APC = − 5.29, *p* < 0.05). However, it then displayed a gradually increasing trend (APC = 0.96, *p* = 0.167) (Supplementary Figure S1). Over the 16-year study period, the 30-day mortality risk after hip fracture declined among women from 3.21 in 2000 to 2.27 in 2015, whereas men experienced a decrease from 4.57 in 2000 to 3.98 in 2015. However, males had a consistently higher 30-day mortality risk than females each year (Fig. 2a). We used the annual SMR to compare the 30-day mortality of hip fractures with that of the general population, and we found that it decreased from 1.71 to 1.65 overall and went from 1.79 to 1.62 in women. However, the annual SMR for males increased from 1.75 to 1.83 (Tables 2 and 3). Males had a higher mortality risk than females across all age groups, and their mortality risk increased with age (Fig. 2b).

**Table 2:** Crude 30-day mortality and standardized mortality ratio (SMR) in patients with hip fracture by year.

	Hip fractures	Deaths	Crude MR (%)	SMR	95% CI
2000	14,961	563	3.76	1.71	1.57–1.85
2001	15,224	509	3.34	1.54	1.41–1.68
2002	15,536	539	3.47	1.64	1.51–1.78
2003	16,267	545	3.35	1.61	1.47–1.74
2004	16,932	525	3.10	1.50	1.38–1.63
2005	17,387	454	2.61	1.27	1.15–1.39
2006	16,847	462	2.74	1.43	1.30–1.56
2007	17,329	503	2.90	1.50	1.37–1.63
2008	18,394	512	2.78	1.45	1.32–1.57
2009	18,626	496	2.66	1.43	1.31–1.56
2010	18,769	493	2.63	1.43	1.30–1.55
2011	19,432	546	2.81	1.50	1.37–1.62
2012	19,686	586	2.98	1.61	1.48–1.74
2013	19,902	573	2.88	1.59	1.46–1.72
2014	20,505	613	2.99	1.61	1.48–1.74
2015	20,094	586	2.92	1.65	1.52–1.78

*MR*  mortality risk (per 100 people), *SMR* standardized mortality ratio.

**Table 3:** Crude 30-day mortality and standardized mortality ratio (SMR) in patients with hip fracture by sex and year.

	Hip fractures	Deaths	Crude MR (%)	SMR	95% CI
Women					
2000	8828	283	3.21	1.79	1.58–2.00
2001	8903	226	2.54	1.46	1.27–1.65
2002	9252	264	2.85	1.67	1.47–1.87
2003	9611	255	2.65	1.57	1.37–1.76
2004	10,038	233	2.32	1.41	1.23–1.60
2005	10,319	210	2.04	1.26	1.09–1.43
2006	10,007	235	2.35	1.56	1.36–1.75
2007	10,445	235	2.25	1.46	1.28–1.65
2008	11,075	254	2.29	1.52	1.33–1.70
2009	11,237	242	2.15	1.47	1.28–1.65
2010	11,226	241	2.15	1.48	1.29–1.66
2011	11,726	269	2.29	1.56	1.37–1.74
2012	11,923	268	2.25	1.54	1.35–1.72
2013	12,116	292	2.41	1.68	1.49–1.88
2014	12,669	299	2.36	1.60	1.42–1.78
2015	12,479	283	2.27	1.62	1.43–1.81
Men					
2000	6133	280	4.57	1.75	1.55–1.96
2001	6321	283	4.48	1.73	1.53–1.93
2002	6284	275	4.38	1.74	1.54–1.95
2003	6656	290	4.36	1.76	1.56–1.96
2004	6894	292	4.24	1.71	1.51–1.90
2005	7068	244	3.45	1.38	1.21–1.56
2006	6840	227	3.32	1.42	1.23–1.60
2007	6884	268	3.89	1.66	1.46–1.86
2008	7319	258	3.53	1.50	1.32–1.68
2009	7389	254	3.44	1.51	1.33–1.70
2010	7543	252	3.34	1.48	1.30–1.67
2011	7706	277	3.60	1.56	1.38–1.74
2012	7763	318	4.10	1.81	1.61–2.01
2013	7786	281	3.61	1.62	1.43–1.81
2014	7836	314	4.01	1.77	1.57–1.97
2015	7615	303	3.98	1.83	1.63–2.04

*MR* mortality risk (per 100 people), *SMR* standardized mortality ratio.

### Risk factors for hip fracture mortality

 The multivariate Cox proportional hazards analysis revealed independent risk factors for hip fracture mortality. These included older age, male sex, higher CCI, residing in suburban, rural, or remote areas, and lower income level (Table 4).

**Table 4:** Hazard ratios for 30–days mortality after hip fracture based on multivariable cox regression.

	HR (95% CI)	*p* value	aHR (95% CI)	*p* value
Gender				
Female	ref.		ref.	
Male	1.64 (1.57–1.71)	< 0.001	1.74 (1.66–1.81)	< 0.001
Age				
50–54	ref.		ref.	
55–59	1.11 (0.86–1.44)	0.409	1.10 (0.85–1.42)	0.485
60–64	1.20 (0.94–1.52)	0.145	1.14 (0.90–1.45)	0.279
65–69	1.70 (1.36–2.12)	< 0.001	1.62 (1.30–2.02)	< 0.001
70–74	1.83 (1.48–2.26)	< 0.001	1.73 (1.40–2.14)	< 0.001
75–79	2.34 (1.91–2.88)	< 0.001	2.21 (1.80–2.72)	< 0.001
80–84	2.90 (2.37–3.55)	< 0.001	2.77 (2.26–3.40)	< 0.001
≧ 85	5.01 (4.10–6.12)	< 0.001	5.05 (4.13–6.18)	< 0.001
CCI				
0	ref.		ref.	
1–3	1.35 (1.25–1.45)	< 0.001	1.24 (1.16–1.34)	< 0.001
4–5	1.80 (1.66–1.94)	< 0.001	1.62 (1.50–1.76)	< 0.001
≧6	2.55 (2.36–2.75)	< 0.001	2.40 (2.22–2.59)	< 0.001
Place of residence				
Urban	ref.		ref.	
Suburban	1.15 (1.09–1.20)	< 0.001	1.16 (1.10–1.22)	< 0.001
Rural or remote area	1.28 (1.21–1.35)	< 0.001	1.27 (1.19–1.35)	< 0.001
Income level				
Low	ref.		ref.	
Intermediate	0.99 (0.94–1.03)	0.527	1.07 (1.01–1.12)	0.013
High	0.79 (0.72–0.87)	< 0.001	0.88 (0.80–0.96)	0.007

*HR*  hazard ratio, *aHR*  adjusted hazard ratio, *CCI* Charlson comorbidity index.

### Significant causes of death after hip fracture

**Fig. 3:**
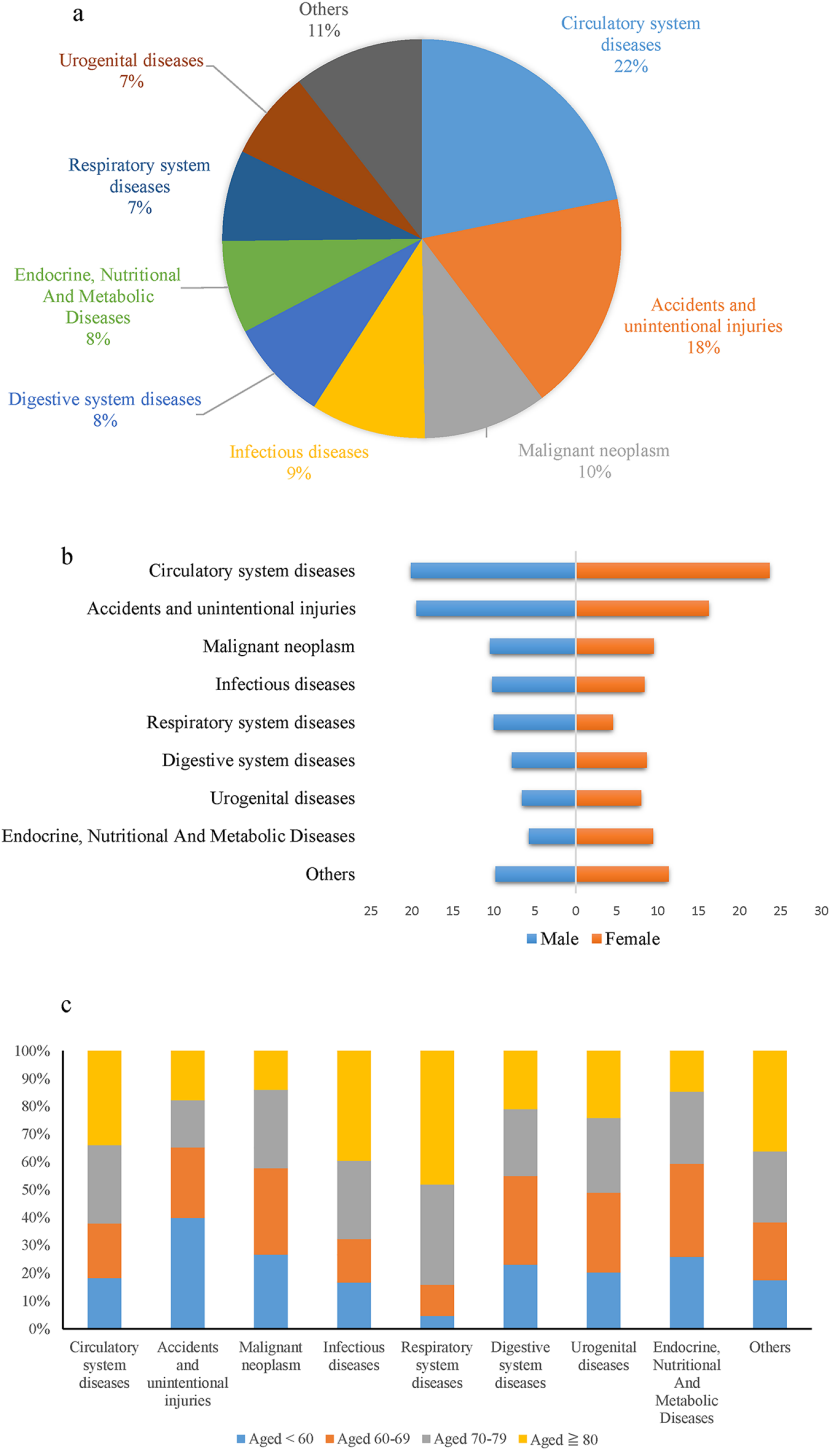
The proportional distribution of the major causes of death among hip fracture patients. **a** Overall population; **b** by sex; **c** by age group.

The pie chart depicted in Fig. 3a provides an overview of the percentage of individuals who died within 30 days after sustaining a hip fracture. During this period, circulatory system diseases were the primary cause of death, accounting for 22% of all cases. Accidents and unintentional injuries closely followed at 18%, malignant neoplasms accounted for 10%, and infectious diseases accounted for 9%. Among infectious diseases, pneumonia was the most prevalent, accounting for 64.11% of infectious disease-related deaths (594 out of 794). When examining the data by sex, circulatory system diseases were the leading cause of death among women. Men had a higher incidence of mortality due to circulatory system diseases and accidents/unintentional injuries (Fig. 3b). Analyzing the data by age group, accidents and unintentional injuries (26.81%) were the leading causes of death among individuals younger than 70. Conversely, circulatory system diseases (22.78%) were the predominant cause of mortality among those aged 70 years and older (Fig. 3c). Furthermore, when stratifying by age and sex, similar proportional distributions of the significant causes of death were observed (Supplementary Figure S2).

## Discussion

We assessed the factors associated with mortality and specific causes of death within 30 days after hip fracture hospitalization. We observed a decrease in the 30-day mortality risk from 3.76 per 100 people in 2000 to 2.92 per 100 people in 2015. Men consistently had a higher 30-day mortality risk than women across all age groups, and their mortality risk increased with age. We also found that older age, male sex, higher CCI, residing in suburban, rural, or remote areas, and lower income level were risk factors for hip fracture mortality. Furthermore, circulatory system diseases, especially ischemic heart disease and cerebrovascular diseases, were the leading cause of death within 30 days after hip fracture, followed by accidents and unintentional injuries, malignant neoplasms, and infectious diseases, with pneumonia being the most common among the latter.

Our findings are consistent with previous studies, which have indicated that circulatory system diseases like ischemic heart disease and cerebrovascular disease are common causes of death within 30 days after hip fracture^25,26^. This may be because patients with circulatory system diseases, particularly those with frailty, are more susceptible to falls due to impaired blood circulation, which increases the risk of fragility fractures such as hip fractures^27–29^. Consequently, this elevated fracture risk may also increase the likelihood of mortality after fracture^30^.

Additionally, previous studies have indicated that patients with a history of stroke have a greater risk of postoperative complications following hip fracture^31,32^. Patients with stroke typically exhibit a higher prevalence of unfavorable prognostic factors, such as postoperative delirium, nutritional problems, and impaired function. These factors collectively contribute to increased mortality rates. The significant impact of circulatory system diseases on early mortality after hip fracture has crucial clinical implications. Combining this knowledge with an understanding of factors associated with early mortality, high-risk patients could be identified and provided early multidisciplinary interventions.

Pneumonia is a common cause of death and is a preventable factor in patients with hip fractures^14,33^. It can be classified as either bacterial pneumonia or aspiration pneumonia. Maintaining oral hygiene, quitting smoking, and receiving influenza or pneumococcal vaccinations can prevent bacterial pneumonia^34^. Aspiration pneumonia is also prevalent in patients with hip fractures, often accompanied by dysphagia and weakness^35^. Improving swallowing postural training and preventing gastroesophageal reflux in patient education can reduce the occurrence of pneumonia^36^. Therefore, unless clinically unstable, patients with a history of pneumonia should not have their surgery delayed. Additionally, early initiation of antibiotic treatment and postoperative pulmonary rehabilitation programs are recommended in these patients^37^.

Previous studies have shown that men have a higher mortality rate than women after hip fracture^38,39^, which is supported by our findings. However, the exact reasons for this sex difference remain unclear^40^. There are several possible explanations for this disparity. First, men may be sicker and frailer than women when fractures occur^40^. Second, males with hip fractures often have poorer bone health (underdiagnosed and undertreated osteoporosis), which contributes to the increased mortality risk^41^. Third, previous research has shown that men with hip fractures have a higher incidence of complications than women. This difference in outcomes has been linked to the higher prevalence and severity of preexisting chronic diseases among men with hip fractures. These underlying health conditions may increase the risk of postoperative complications in male patients^11,42^. Furthermore, male hip fracture patients show a more profound impairment in daily activities, functional capacity, and walking speed than women. Therefore, men may experience a greater physiological reserve loss after hip fracture, leading to a higher mortality risk^43^.

Our findings that residents of non-urban areas have a higher mortality rate following hip fractures are consistent with the results of other studies^44–46^. This increased mortality may be due to several contributing factors. Limited access to healthcare services and medical professionals in rural areas can delay treatment, leading to a higher risk of complications and death. Moreover, lower income levels may restrict access to quality care, while inadequate management of chronic conditions, such as cardiovascular disease, can further increase mortality risk^47^. The geographic distance to medical centers and the resulting delays in receiving care, as well as reduced social support during recovery, are additional challenges that likely contribute to the elevated mortality rate observed in rural populations.

We identified sex-based differences in the causes of death following hip fracture. Our findings revealed that men were more likely than women to die from respiratory diseases, which is potentially attributed to the higher prevalence of smoking among men, as suggested by a previous study in Taiwan^48^. This may explain the higher mortality rate observed in men, because they are more susceptible to experiencing exacerbated respiratory issues after hip fracture surgery, including reduced airway secretions, impaired daily activity, and subsequent chest complications. We also found that men had a higher mortality rate due to infectious diseases than women. This disparity may be linked to the age-related decline in immune function, which can increase susceptibility to infections among the elderly^49^. Several studies have highlighted male sex as a risk factor for significant infections in postfracture populations^50,51^, with male patients in surgical intensive care units having a higher incidence of severe sepsis or septic shock^52^, leading to an elevated mortality risk from infections. Moreover, accidents and unintentional injuries also contribute to the higher mortality rate among men. This may be because, in Taiwan, males more often drive and ride motorcycles than women, increasing their risk of vehicle and traffic-related accidents, potentially leading to a higher mortality rate^53^.

Our data show a 30-day mortality rate of 2.97%, consistent with previous reports from Taiwan^54^ and comparable to other Asian countries^55^. Japan (1.2%), Thailand (1.2%), South Korea (1.9%), Singapore (2.2%), China (3.3%), and Hong Kong SAR (3.0%) report rates between 1.2% and 3.3%^55^, which are notably lower than those in England (9.6%), Scotland (7%), and the US (5.2–9.3%)^9,56–58^. This lower mortality in Asia may be influenced by healthier diets, lower obesity rates, and better healthcare access through national insurance systems, enabling timely treatment. Earlier surgical intervention and comprehensive post-operative care, along with cultural factors like stronger family support and more active elderly lifestyles, also likely contribute to better outcomes. Previous studies have reported a declining trend in the 30-day mortality rate^26,58^, whereas a study from Sweden showed the opposite trend^59^. However, our study observed a declining trend followed by a plateau. The exact reasons for this change in the mortality rate remain unclear. The decrease in mortality rates before 2006 may be attributed to the launch in 1995 of the national insurance program, which improved healthcare services in Taiwan. Additionally, advances in surgical techniques and the wide-scale implementation of the case payment system by Taiwan’s health insurance program in 2002 provided better funding and more comprehensive care for patients with hip fracture. These factors may have contributed to the stable mortality rates observed after 2006.

The strengths of this study include its large sample size derived from a national database, a long follow-up period, and its focus on a topic with relatively few existing studies. This underscores the significant opportunities for prevention and intervention. However, some limitations should be noted. Our study relied on national claims data, which did not allow us to evaluate the impact of factors such as body mass index, smoking history, alcohol abuse, nutritional status, surgery-related information, and postoperative health status on the 30-day mortality rate after hip fracture. Additionally, the expiration of our database access rights prevented us from analyzing the specific causes of hip fractures and assessing how different factors, such as fragility fractures versus fractures caused by metastases or traffic accidents, might influence mortality rates.

## Conclusion and future directions

Our research indicates that there has been a declining trend in the 30-day mortality rate after hip fracture in Taiwan. We observed a significant correlation between the 30-day mortality rate and male sex, increasing age, the presence of comorbidities, place of residence, and lower income. The primary causes of death within 30 days were circulatory system diseases, accidents and unintentional injuries, malignant tumors, and infectious diseases. Early identification of high-risk patients is crucial for implementing targeted interventions to reduce in-hospital complications. Since patients with hip fracture may have limited physiological reserves, these patients should receive treatment from experienced clinicians. Identifying and addressing their specific needs can optimize outcomes and minimize complications during hospitalization, thereby reducing mortality rates.

## Electronic supplementary material

Below is the link to the electronic supplementary material.

Supplementary Material 1

Supplementary Material 2

Supplementary Material 3

Supplementary Material 4

## Data Availability

Data Availability StatementData from the National Health Insurance Research Database, administered by Taiwan’s Ministry of Health and Welfare (MOHW), are subject to stringent data protection laws and cannot be openly disclosed. Requests for data access must adhere to a formal application process outlined at http://dep.mohw.gov.tw. Please contact the staff of MOHW (Email: stdlwu@mohw.gov.tw) for assistance.

## Abbreviations

APC: Annual percentage change
CCI: Charlson Comorbidity Index
ICD-9-CM: International Classification of Diseases, Ninth Revision, Clinical Modification
NHIRD: National Health Insurance Research Database
NTD: New Taiwan dollars
SMRs: Standardized mortality ratios

## References

[1] Kanis, J. A. et al. A systematic review of hip fracture incidence and probability of fracture worldwide. *Osteoporos. Int.* **23**, 2239–2256. 10.1007/s00198-012-1964-3 (2012). 22419370 10.1007/s00198-012-1964-3 PMC3421108

[2] Gullberg, B., Johnell, O. & Kanis, J. A. World-wide projections for hip fracture. *Osteoporos. Int.* **7**, 407–413. 10.1007/pl00004148 (1997). 9425497 10.1007/pl00004148

[3] Giannoulis, D., Calori, G. M. & Giannoudis, P. V. Thirty-day mortality after hip fractures: has anything changed? *Eur. J. Orthop. Surg. Traumatol.* **26**, 365–370. 10.1007/s00590-016-1744-4 (2016). 26943870 10.1007/s00590-016-1744-4 PMC4856719

[4] Schnell, S., Friedman, S. M., Mendelson, D. A., Bingham, K. W. & Kates, S. L. The 1-year mortality of patients treated in a hip fracture program for elders. *Geriatr. Orthop. Surg. Rehabil*. **1**, 6–14. 10.1177/2151458510378105 (2010). 23569656 10.1177/2151458510378105 PMC3597289

[5] Hu, F., Jiang, C., Shen, J., Tang, P. & Wang, Y. Preoperative predictors for mortality following hip fracture surgery: A systematic review and meta-analysis. *Injury*. **43**, 676–685. 10.1016/j.injury.2011.05.017 (2012). 21683355 10.1016/j.injury.2011.05.017

[6] Xu, B. Y., Yan, S., Low, L. L., Vasanwala, F. F. & Low, S. G. Predictors of poor functional outcomes and mortality in patients with hip fracture: A systematic review. *BMC Musculoskelet. Disord*. **20**, 568. 10.1186/s12891-019-2950-0 (2019). 31775693 10.1186/s12891-019-2950-0 PMC6882152

[7] Karres, J., Zwiers, R., Eerenberg, J. P., Vrouenraets, B. C. & Kerkhoffs, G. Mortality prediction in hip fracture patients: Physician assessment versus prognostic models. *J. Orthop. Trauma.* **36**, 585–592. 10.1097/BOT.0000000000002412 (2022). 35605101 10.1097/BOT.0000000000002412 PMC9555757

[8] Barrett, J. A., Baron, J. A. & Beach, M. L. Mortality and pulmonary embolism after fracture in the elderly. *Osteoporos. Int.* **14**, 889–894. 10.1007/s00198-003-1494-0 (2003). 12942231 10.1007/s00198-003-1494-0

[9] Roche, J. J., Wenn, R. T., Sahota, O. & Moran, C. G. Effect of comorbidities and postoperative complications on mortality after hip fracture in elderly people: prospective observational cohort study. *BMJ*. **331**, 1374. 10.1136/bmj.38643.663843.55 (2005). 16299013 10.1136/bmj.38643.663843.55 PMC1309645

[10] Sathiyakumar, V. et al. Risk factors for adverse cardiac events in hip fracture patients: An analysis of NSQIP data. *Int. Orthop.* **40**, 439–445. 10.1007/s00264-015-2832-5 (2016). 26194916 10.1007/s00264-015-2832-5

[11] Kannegaard, P. N., van der Mark, S., Eiken, P. & Abrahamsen, B. Excess mortality in men compared with women following a hip fracture. National analysis of comedications, comorbidity and survival. *Age Ageing*. **39**, 203–209. 10.1093/ageing/afp221 (2010). 20075035 10.1093/ageing/afp221

[12] Abtahi, S. et al. The association of oral bisphosphonate use with mortality risk following a major osteoporotic fracture in the United Kingdom: Population-based Cohort Study. *J. Am. Med. Dir. Assoc.* **21**, 811–816. 10.1016/j.jamda.2019.11.003 (2020). 31839557 10.1016/j.jamda.2019.11.003

[13] Deakin, D. E., Boulton, C. & Moran, C. G. Mortality and causes of death among patients with isolated limb and pelvic fractures. *Injury*. **38**, 312–317. 10.1016/j.injury.2006.09.024 (2007). 17141780 10.1016/j.injury.2006.09.024

[14] Sheikh, H. Q. et al. A comprehensive analysis of the causes and predictors of 30-Day mortality following hip fracture surgery. *Clin. Orthop. Surg.* **9**, 10–18. 10.4055/cios.2017.9.1.10 (2017). 28261422 10.4055/cios.2017.9.1.10 PMC5334018

[15] Groff, H. et al. Causes of in-hospital mortality after hip fractures in the elderly. *Hip Int.* **30**, 204–209. 10.1177/1120700019835160 (2020). 30909746 10.1177/1120700019835160

[16] Sennerby, U. et al. Cardiovascular diseases and risk of hip fracture. *JAMA*. **302**, 1666–1673. 10.1001/jama.2009.1463 (2009). 19843901 10.1001/jama.2009.1463

[17] Tsai, Y. L. et al. Drug adherence and treatment duration for denosumab and mortality risk among hip fracture patients. *Osteoporos. Int.*10.1007/s00198-023-06845-0 (2023). 37466659 10.1007/s00198-023-06845-0

[18] Lee, M. T. et al. Epidemiology and clinical impact of osteoporosis in Taiwan: A 12-year trend of a nationwide population-based study. *J. Formos. Med. Assoc.*10.1016/j.jfma.2023.05.001 (2023). 37208247 10.1016/j.jfma.2023.05.001

[19] Lee, M. S., Yeh, Y. C., Chang, Y. T. & Lai, M. S. All-cause and cause-specific mortality in patients with psoriasis in Taiwan: A Nationwide Population-based study. *J. Investig. Dermatol.* **137**, 1468–1473. 10.1016/j.jid.2017.01.036 (2017). 28257796 10.1016/j.jid.2017.01.036

[20] Lu, C. L., Chang, Y. H., Martini, S., Chang, M. F. & Li, C. Y. Overall and cause-specific mortality in patients with type 1 diabetes Mellitus: A Population-based Cohort Study in Taiwan from 1998 through 2014. *J. Epidemiol.* **31**, 503–510. 10.2188/jea.JE20200026 (2021). 32741854 10.2188/jea.JE20200026 PMC8328860

[21] Lu, T. H., Lee, M. C. & Chou, M. C. Accuracy of cause-of-death coding in Taiwan: Types of miscoding and effects on mortality statistics. *Int. J. Epidemiol.* **29**, 336–343. 10.1093/ije/29.2.336 (2000). 10817134 10.1093/ije/29.2.336

[22] Charlson, M. E., Pompei, P., Ales, K. L. & MacKenzie, C. R. A new method of classifying prognostic comorbidity in longitudinal studies: Development and validation. *J. Chronic Dis.* **40**, 373–383. 10.1016/0021-9681(87)90171-8 (1987). 3558716 10.1016/0021-9681(87)90171-8

[23] Liu, C. Y. et al. Incorporating development stratification of Taiwan townships into sampling design of large scale health interview survey. *J. Health Manag.* **1**, 1–22 (2006).

[24] Kim, H. J., Fay, M. P., Feuer, E. J. & Midthune, D. N. Permutation tests for joinpoint regression with applications to cancer rates. *Stat. Med.* **19**, 335–351. https://doi.org/10.1002/(sici)1097-0258(20000215)19:3<335::aid-sim336>3.0.co;2-z (2000). 10.1002/(sici)1097-0258(20000215)19:3<335::aid-sim336>3.0.co;2-z 10649300

[25] Holvik, K. et al. Cause-specific excess mortality after hip fracture: The Norwegian epidemiologic osteoporosis studies (NOREPOS). *BMC Geriatr.* **23**, 201. 10.1186/s12877-023-03910-5 (2023). 36997876 10.1186/s12877-023-03910-5 PMC10064567

[26] Chatterton, B. D., Moores, T. S., Ahmad, S., Cattell, A. & Roberts, P. J. Cause of death and factors associated with early in-hospital mortality after hip fracture. *Bone Jt. J.* **97-B**, 246–251. 10.1302/0301-620X.97B2.35248 (2015). 10.1302/0301-620X.97B2.35248 25628290

[27] Vestergaard, P., Rejnmark, L. & Mosekilde, L. Increased mortality in patients with a hip fracture-effect of pre-morbid conditions and post-fracture complications. *Osteoporos. Int.* **18**, 1583–1593. 10.1007/s00198-007-0403-3 (2007). 17566814 10.1007/s00198-007-0403-3

[28] Heidari, S. M., Soltani, H., Hashemi, S. J., Talakoub, R. & Soleimani, B. Comparative study of two anesthesia methods according to postoperative complications and one month mortality rate in the candidates of hip surgery. *J. Res. Med. Sci.* **16**, 323–330 (2011). 22091252 PMC3214341

[29] Lawrence, V. A., Hilsenbeck, S. G., Noveck, H., Poses, R. M. & Carson, J. L. Medical complications and outcomes after hip fracture repair. *Arch. Intern. Med.* **162**, 2053–2057. 10.1001/archinte.162.18.2053 (2002). 12374513 10.1001/archinte.162.18.2053

[30] Kilci, O. et al. Postoperative mortality after hip fracture surgery: A 3 years follow up. *PLoS One*. **11**, e0162097. 10.1371/journal.pone.0162097 (2016). 27788137 10.1371/journal.pone.0162097 PMC5082940

[31] Foster, E. J. et al. Long-term factors associated with falls and fractures poststroke. *Front. Neurol.* **9**, 210. 10.3389/fneur.2018.00210 (2018). 29666603 10.3389/fneur.2018.00210 PMC5891595

[32] Wei, W. E. et al. Post-stroke patients with moderate function have the greatest risk of falls: A National Cohort Study. *BMC Geriatr.* **19**, 373. 10.1186/s12877-019-1377-7 (2019). 31878876 10.1186/s12877-019-1377-7 PMC6933903

[33] Jang, S. Y. et al. Effect of pneumonia on all-cause mortality after elderly hip fracture: A Korean Nationwide Cohort Study. *J. Korean Med. Sci.* **35**, e9. 10.3346/jkms.2020.35.e9 (2020). 31920015 10.3346/jkms.2020.35.e9 PMC6955432

[34] Tanzella, G., Motos, A., Battaglini, D., Meli, A. & Torres, A. Optimal approaches to preventing severe community-acquired pneumonia. *Expert Rev. Respir. Med.* **13**, 1005–1018. 10.1080/17476348.2019.1656531 (2019). 31414915 10.1080/17476348.2019.1656531

[35] Reza Shariatzadeh, M., Huang, J. Q. & Marrie, T. J. Differences in the features of aspiration pneumonia according to site of acquisition: Community or continuing care facility. *J. Am. Geriatr. Soc.* **54**, 296–302. 10.1111/j.1532-5415.2005.00608.x (2006). 16460382 10.1111/j.1532-5415.2005.00608.x

[36] Baijens, L. W. et al. European Society for Swallowing Disorders—European Union Geriatric Medicine Society white paper: Oropharyngeal dysphagia as a geriatric syndrome. *Clin. Interv Aging*. **11**, 1403–1428. 10.2147/CIA.S107750 (2016). 27785002 10.2147/CIA.S107750 PMC5063605

[37] Chang, S. C. et al. Reduction in the incidence of pneumonia in elderly patients after hip fracture surgery: An inpatient pulmonary rehabilitation program. *Medicine (Baltimore)* **97**, e11845. 10.1097/MD.0000000000011845 (2018). 30113476 10.1097/MD.0000000000011845 PMC6113002

[38] Kjaervik, C., Gjertsen, J. E., Stensland, E., Saltyte-Benth, J. & Soereide, O. Modifiable and non-modifiable risk factors in hip fracture mortality in Norway, 2014 to 2018: A linked multiregistry study. *Bone Jt. J.* **104-B**, 884–893. 10.1302/0301-620X.104B7.BJJ-2021-1806.R1 (2022). 10.1302/0301-620X.104B7.BJJ-2021-1806.R1 PMC9251134 35775181

[39] Kusen, J. Q. et al. The implementation of a geriatric fracture centre for hip fractures to reduce mortality and morbidity: An observational study. *Arch. Orthop. Trauma. Surg.* **139**, 1705–1712. 10.1007/s00402-019-03229-0 (2019). 31309288 10.1007/s00402-019-03229-0

[40] Poor, G., Atkinson, E. J., O’Fallon, W. M. & Melton, L. J. 3rd. Determinants of reduced survival following hip fractures in men. *Clin. Orthop. Relat. Res.* **319**, 260–265 (1995). 7554638

[41] Sing, C. W. et al. Global epidemiology of hip fractures: Secular trends in incidence rate, post-fracture treatment, and all-cause mortality. *J. Bone Min. Res.* **38**, 1064–1075. 10.1002/jbmr.4821 (2023). 10.1002/jbmr.4821 37118993

[42] Sheehan, K. J., Sobolev, B., Chudyk, A., Stephens, T. & Guy, P. Patient and system factors of mortality after hip fracture: A scoping review. *BMC Musculoskelet. Disord*. **17**, 166. 10.1186/s12891-016-1018-7 (2016). 27079195 10.1186/s12891-016-1018-7 PMC4832537

[43] Fransen, M. et al. Excess mortality or institutionalization after hip fracture: Men are at greater risk than women. *J. Am. Geriatr. Soc.* **50**, 685–690. 10.1046/j.1532-5415.2002.50163.x (2002). 11982669 10.1046/j.1532-5415.2002.50163.x

[44] Hsu, I. L. et al. Socioeconomic inequality in one-year mortality of Elderly people with hip fracture in Taiwan. *Int. J. Environ. Res. Public. Health*. **15**10.3390/ijerph15020352 (2018). 10.3390/ijerph15020352 PMC5858421 29462914

[45] Farley, B. J. et al. Rural, urban, and teaching hospital differences in hip fracture mortality. *J. Orthop.* **21**, 453–458. 10.1016/j.jor.2020.08.039 (2020). 32982100 10.1016/j.jor.2020.08.039 PMC7494603

[46] Diamantopoulos, A. P., Hoff, M., Skoie, I. M., Hochberg, M. & Haugeberg, G. Short- and long-term mortality in males and females with fragility hip fracture in Norway. A population-based study. *Clin. Interv Aging*. **8**, 817–823. 10.2147/CIA.S45468 (2013). 23861581 10.2147/CIA.S45468 PMC3704300

[47] Kristensen, P. K., Thillemann, T. M., Pedersen, A. B., Soballe, K. & Johnsen, S. P. Socioeconomic inequality in clinical outcome among hip fracture patients: A nationwide cohort study. *Osteoporos. Int.* **28**, 1233–1243. 10.1007/s00198-016-3853-7 (2017). 27909785 10.1007/s00198-016-3853-7

[48] Panula, J. et al. Mortality and cause of death in hip fracture patients aged 65 or older: A population-based study. *BMC Musculoskelet. Disord*. **12**, 105. 10.1186/1471-2474-12-105 (2011). 21599967 10.1186/1471-2474-12-105 PMC3118151

[49] Yung, R. L. Changes in immune function with age. *Rheum. Dis. Clin. N. Am.* **26**, 455–473. 10.1016/s0889-857x(05)70151-4 (2000). 10.1016/s0889-857x(05)70151-4 10989507

[50] Wehren, L. E. et al. Gender differences in mortality after hip fracture: The role of infection. *J. Bone Min. Res.* **18**, 2231–2237. 10.1359/jbmr.2003.18.12.2231 (2003). 10.1359/jbmr.2003.18.12.2231 14672359

[51] Chou, F. P. et al. Sex differences in fracture outcomes within Taiwan population: A nationwide matched study. *PLoS One*. **15**, e0231374. 10.1371/journal.pone.0231374 (2020). 32271850 10.1371/journal.pone.0231374 PMC7144979

[52] Wichmann, M. W., Inthorn, D., Andress, H. J. & Schildberg, F. W. Incidence and mortality of severe sepsis in surgical intensive care patients: The influence of patient gender on disease process and outcome. *Intensive Care Med.* **26**, 167–172. 10.1007/s001340050041 (2000). 10784304 10.1007/s001340050041

[53] Pan, R. H. et al. Epidemiology of orthopedic fractures and other injuries among inpatients admitted due to traffic accidents: A 10-year nationwide survey in Taiwan. *Sci. World J.* 637872 (2014). 10.1155/2014/637872 (2014). 10.1155/2014/637872 PMC3932229 24672344

[54] Shao, C. J., Hsieh, Y. H., Tsai, C. H. & Lai, K. A. A nationwide seven-year trend of hip fractures in the elderly population of Taiwan. *Bone*. **44**, 125–129. 10.1016/j.bone.2008.09.004 (2009). 18848656 10.1016/j.bone.2008.09.004

[55] Harvey, L. A. et al. Variation in mortality following hip fracture across the Asia Pacific region: Systematic review and proportional meta-analysis. *Arch. Gerontol. Geriatr.* **126**, 105519. 10.1016/j.archger.2024.105519 (2024). 38941947 10.1016/j.archger.2024.105519

[56] Holt, G., Smith, R., Duncan, K., Finlayson, D. F. & Gregori, A. Early mortality after surgical fixation of hip fractures in the elderly: An analysis of data from the Scottish hip fracture audit. *J. Bone Joint Surg. Br.* **90**, 1357–1363. 10.1302/0301-620X.90B10.21328 (2008). 18827248 10.1302/0301-620X.90B10.21328

[57] Bass, E., French, D. D., Bradham, D. D. & Rubenstein, L. Z. Risk-adjusted mortality rates of elderly veterans with hip fractures. *Ann. Epidemiol.* **17**, 514–519. 10.1016/j.annepidem.2006.12.004 (2007). 17420142 10.1016/j.annepidem.2006.12.004

[58] Brauer, C. A., Coca-Perraillon, M., Cutler, D. M. & Rosen, A. B. Incidence and mortality of hip fractures in the United States. *JAMA*. **302**, 1573–1579. 10.1001/jama.2009.1462 (2009). 19826027 10.1001/jama.2009.1462 PMC4410861

[59] Nordstrom, P., Bergman, J., Ballin, M. & Nordstrom, A. Trends in Hip fracture incidence, length of Hospital Stay, and 30-Day mortality in Sweden from 1998–2017: A Nationwide Cohort Study. *Calcif Tissue Int.* **111**, 21–28. 10.1007/s00223-022-00954-4 (2022). 35166892 10.1007/s00223-022-00954-4 PMC9232476

