# Supplementary figures and images for: Exploring mortality risk factors and specific causes of death within 30 days after hip fracture hospitalization
Source: Sci Rep. 2024 Nov 11;14:27544. doi: 10.1038/s41598-024-79297-z (PMC11555071; doi:10.1038/s41598-024-79297-z)

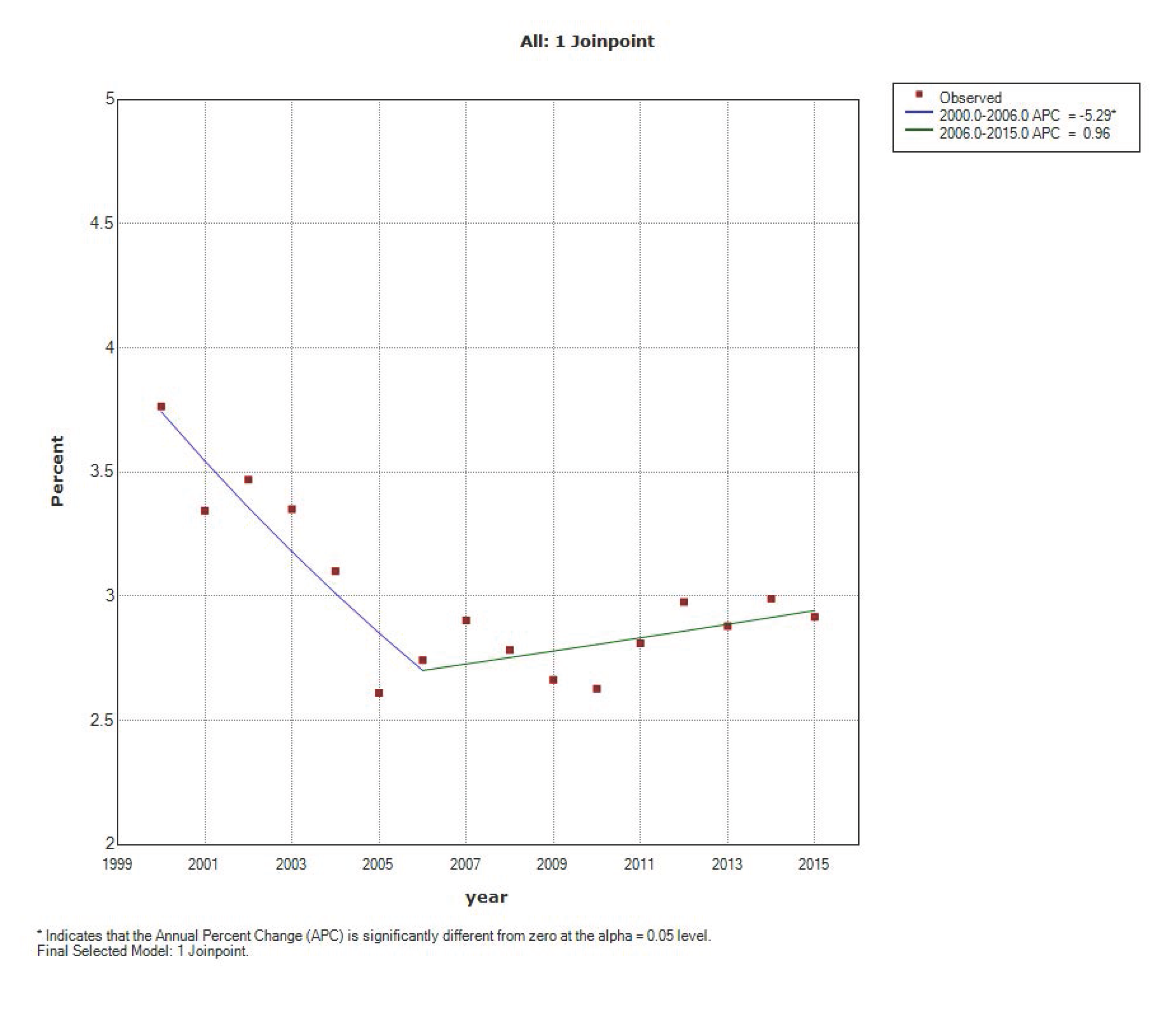

Supplement: Supplementary file 1 — Supplementary Material 1 [file 41598_2024_79297_MOESM1_ESM.tif]

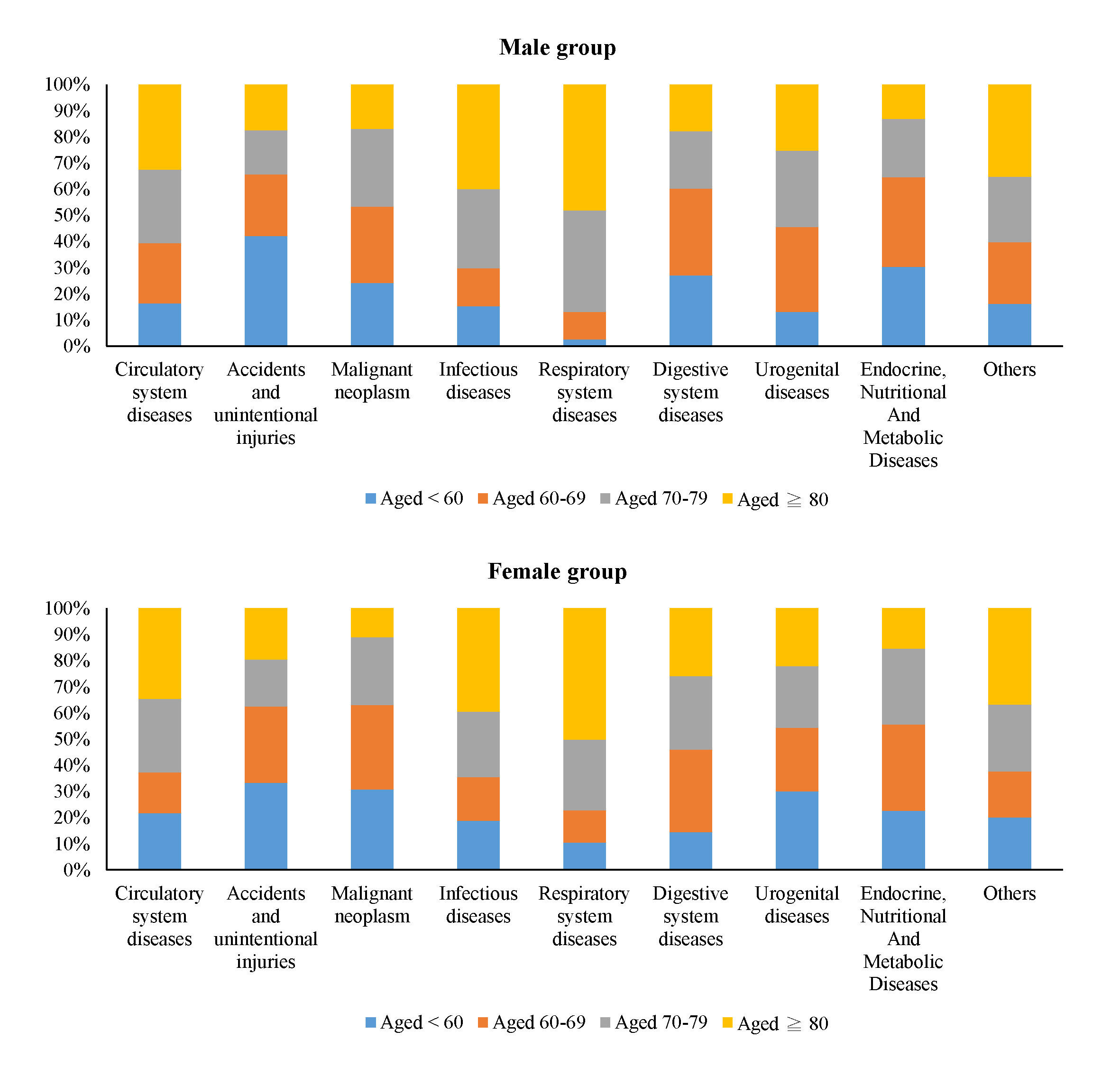

Supplement: Supplementary file 2 — Supplementary Material 2 [file 41598_2024_79297_MOESM2_ESM.tiff]
