# Supplementary material for: Exploring mortality risk factors and specific causes of death within 30 days after hip fracture hospitalization
Source: Sci Rep. 2024 Nov 11;14:27544. doi: 10.1038/s41598-024-79297-z (PMC11555071; doi:10.1038/s41598-024-79297-z)
Supplement: Supplementary file 3 — Supplementary Material 3 [file 41598_2024_79297_MOESM3_ESM.docx]

**SUPPLEMENTARY DATA**

**Exploring mortality risk factors and specific causes of death within 30 days after hip fracture hospitalization**

Cheng-Yi Wu^1,2^, Ching-Fang Tsai^3^, Yueh-Han Hsu^4, 5, 6^, Hsin-Yi Yang^3*^

^1^Department of Orthopedics, Ditmanson Medical Foundation Chia-Yi Christian Hospital, Chiayi City 600, Taiwan

^2^ Osteoporosis Center, Ditmanson Medical Foundation Chia-Yi Christian Hospital, Chiayi City 600, Taiwan

^3^ Clinical Data Center, Ditmanson Medical Foundation Chia-Yi Christian Hospital, Chiayi City 600, Taiwan

^4^Department of Medical Research, China Medical University Hospital and China Medical University, Taichung 404, Taiwan

^5^Department of Internal Medicine, Division of Nephrology, Ditmanson Medical Foundation Chia-Yi Christian Hospital, Chiayi City 600, Taiwan

^6^Department of Nursing, Min-Hwei College of Health Care Management, Tainan 736, Taiwan

^*^Correspondent author

Address correspondence and reprint requests to Dr. Hsin-Yi Yang, Clinical Data Center, Ditmanson Medical Foundation Chia-Yi Christian Hospital, No. 539, Zhongxiao Rd., East District, Chiayi City 600, Taiwan. Tel: +886-5-2765041 ext. 5563, E-mail: [cych13018@gmail.com](mailto:cych13018@gmail.com)

**Supplemental Figure 1.** Trends in 30-day mortality from hip fracture in Taiwan, 2000–2015: a joinpoint regression analysis. APC = Annual percent change.

**Supplemental Figure 2.** Distribution of deaths (%) by major cause of death categories within 30 days after hip fracture, differentiated by age group: (a) men; (b) women.

**Supplemental Table 1.** ICD-9 codes for diseases analyzed in this study.
