# Supplementary material for: Exploring mortality risk factors and specific causes of death within 30 days after hip fracture hospitalization
Source: Sci Rep. 2024 Nov 11;14:27544. doi: 10.1038/s41598-024-79297-z (PMC11555071; doi:10.1038/s41598-024-79297-z)
Supplement: Supplementary file 4 — Supplementary Material 4 [file 41598_2024_79297_MOESM4_ESM.docx]

sTable 1. ICD codes for diseases analyzed in this study

| Causes of death | ICD-9-CM codes | ICD-10-CM codes |
| --- | --- | --- |
| **Circulatory system diseases** | 390-459 | I00–I99 |
| Hypertensive diseases | 401-405 | I10–I15 |
| Ischemic heart disease | 410-414 | I20–I25 |
| Diseases of pulmonary circulation | 415–417 | I26-28 |
| Pulmonary embolism | 415.1 | I26 |
| Other forms of heart disease | 420–429 | I30-52 |
| Cerebrovascular diseases | 430-438 | I60-69 |
| Diseases of arteries, arterioles, and capillaries | 440–448 | I70-79 |
| Acute rheumatic fever, chronic rheumatic heart disease, diseases of veins and lymphatics, and other diseases of circulatory system | 390–392; 393–398; 451–459 | I00-08; I09; I80-89 |
| **Malignant neoplasm** | 140-239 | C00-C97 |
| Stomach | 151 | C16 |
| Pancreas | 157 | C25 |
| Liver and intrahepatic bile ducts | 155 | C22 |
| Bronchus and lung | 162 | C34 |
| Colon, rectum, and anus | 153, 154 | C18–C21, C260 |
| Prostate (males only) | 185 | C61 |
| Ovary (females only) | 183 | C56 |
| Bladder | 188 | C67 |
| Breast (females only) | 174 | C50 |
| Kidney | 189 | C64–C65 |
| Cervix uteri (females only) | 180 | C53 |
| **Respiratory system diseases** | 470-478, 490-519 | J30-J99 |
| COPD | 491–496 | J40–J47 |
| **Infectious diseases** | 001-139, 460-466, 480-488 | A00–B99, J00–J22 |
| Sepsis | 038 | A41, R57, R65 |
| Pneumonia | 481, 482, 483, 485, 486 | J12-J18 |
| **Digestive system diseases** | 520-579 | K00–K99 |
| Liver disease | 570-573 | K70-77 |
| Diseases of oral cavity and salivary glands | 520-529 | K00-14 |
| Diseases of esophagus, stomach and duodenum | 530-537 | K20-31 |
| Noninfective enteritis and colitis | 555-558 | K50-52 |
| Other diseases of intestines and appendix | 540-543, 560-566,569 | K35-38, K55-64 |
| Diseases of peritoneum and retroperitoneum | 567-568 | K65-68 |
| Disorders of gallbladder, biliary tract and pancreas | 574-577 | K80-87 |
| Hernia, other diseases of the digestive system | 550-553, 578-579 | K40-46, K90-95 |
| Gastrointestinal bleed | 578 | K92 |

sTable 1. ICD codes for diseases analyzed in this study (continued)

| Causes of death | ICD-9-CM codes | ICD-10-CM codes |
| --- | --- | --- |
| **Urogenital diseases** | 580-629 | N00–N99 |
| Renal diseases | 580-589 | N00-N07, N17-N19, N25-N27 |
| Urinary tract infection | 590, 595, 597, 599.0, 601 | N30, N34, N39 |
| **Endocrine, Nutritional and Metabolic Diseases** | 240-278 | E00–E99 |
| Diabetes | 250 | E10, E11, E14 |
| **Accidents and unintentional injuries** | E800-E949 | V01–X59, Y85–Y86 |
